# SAG-RAD: A Method for Single-Cell Population Genomics of Unicellular Eukaryotes

**DOI:** 10.1093/molbev/msad095

**Published:** 2023-04-20

**Authors:** Raphael Gollnisch, Joel Wallenius, Kristin E Gribble, Dag Ahrén, Karin Rengefors

**Affiliations:** Department of Biology, Aquatic Ecology, Lund University, Lund, Sweden; Department of Earth Sciences, University of Oxford, Oxford, United Kingdom; Faculty of Medicine, Department of Clinical Sciences, Lund University, Lund, Sweden; Marine Biological Laboratory, Woods Hole, MA, USA; National Bioinformatics Infrastructure Sweden (NBIS), SciLifeLab, Department of Biology, Lund University, Lund, Sweden; Department of Biology, Aquatic Ecology, Lund University, Lund, Sweden

**Keywords:** culturing bias, microbial genetic diversity, microeukaryotes, restriction-site associated DNA (RAD) sequencing, single amplified genome (SAG), whole-genome amplification (WGA)

## Abstract

Sequencing of reduced representation libraries enables genotyping of many individuals for population genomic studies. However, high amounts of DNA are required, and the method cannot be applied directly on single cells, preventing its use on most microbes. We developed and implemented the analysis of single amplified genomes followed by restriction-site-associated DNA sequencing to bypass labor-intensive culturing and to avoid culturing bias in population genomic studies of unicellular eukaryotes. This method thus opens the way for addressing important questions about the genetic diversity, gene flow, adaptation, dispersal, and biogeography of hitherto unexplored species.

## Introduction

Single-cell genomics is a transformative research technique that allows resolution of genomic information from the most fundamental unit of life (Lasken and McLean 2014; Gawad et al. 2016). This is particularly true for studies on microorganisms, where individual cells constitute complete organisms. Single-cell genomics has thus become indispensable for population genomic studies of prokaryotes. However, an equivalent method is lacking for unicellular eukaryotes.

Microbial species make up the vast majority of biological diversity on our planet—recent estimates indicate as many as 10^12^ species (Locey and Lennon 2016)—and only a minor fraction can be investigated through traditional culturing-based approaches (Locey and Lennon 2016; Thompson et al. 2017). Therefore, the lack of feasible population genomic methods for these organisms is a major limitation. When microbial culturing can be used, it is time-consuming and costly, success rates are often low, and the limited culturing potential introduces a systematic bias (Snyder et al. 2004; Del Campo et al. 2013) even in species where some individuals grow under artificial laboratory culture conditions. Importantly, strains successfully isolated and cultured must typically go through at least 20 generations before reaching sufficient biomass to enable DNA extraction; this can lead to an accumulation of genomic diversity (Lakeman et al. 2009; Bulankova et al. 2021). There is thus an urgent need to develop alternative genomic approaches for unicellular eukaryotes, to unveil the genomic diversity and function of microbes in nature.

An important alternative approach to culturing is the amplification of individual microbial genomes from single cells using whole-genome amplification (WGA). This method has successfully been used for population genomic studies of the marine cyanobacterium *Prochlorococcus* to sequence thousands of genomes, which has enabled the identification of subpopulations of different ecotypes and separation of core genomes from accessory genomes (Kashtan et al. 2014). However, for a vast number of eukaryotic microorganisms, large genome sizes (e.g., dinoflagellates: 3–215 Gb; Hackett et al. 2005) make it intractable to sequence the number of samples (100s to 1,000s) necessary for population genomic studies.

Instead, for most large-genome eukaryotes, including many bloom-forming phytoplankton, the traditional approach for population genetic studies has relied on culturing, followed by microsatellite or amplified fragment length polymorphism analyses (Rengefors et al. 2017). These methods have limited resolution, preventing the detection of fine-scale patterns in genetic diversity. Additionally, they do not allow coupling of genotype with function, and consequently offer limited information on species ecology and evolution. In contrast, population genomics, the analysis of genome-wide polymorphism data, provides high-resolution information on population genetic structure, selection, and the genetic basis of phenotypes (Ellegren 2014). More recently, reduced representation sequencing (e.g., RADseq) has allowed for population genomic investigations (Rengefors et al. 2021) at a higher resolution in organisms with large genomes. However, genotyping using RADseq requires large amounts of DNA and cannot be used directly on single cells. Individual culturing of clonal strains for each of the hundreds to thousands of samples in population genomic studies is thus still a major bottleneck. Taken together, these issues create a significant methodological gap that prevents population genomic studies of most unicellular eukaryotes.

## New Approaches

Here, we report the conception, realization, and proof-of-principle demonstration of an accurate method for population genomic studies of natural populations of microorganisms with large genomes using a combination of single amplified genomes (SAGs) with reduced representation sequencing (RADseq). Hence, we term this novel approach SAG-RAD, in which established methods to produce SAGs and RADseq libraries are used in tandem in a new application. To achieve optimal genome recovery in SAG-RAD, we optimized crucial aspects of the amplification of single-cell genomes, including lysis conditions and amplification time. To demonstrate the accuracy and versatility of SAG-RAD, we applied our method to *Gonyostomum semen*, a harmful microalga with a large genome (2C ≈ 30 Gb [Rengefors et al. 2021]) that forms nuisance blooms in freshwaters (Rengefors et al. 2021). The method is applicable to other microeukaryotes with large genomes, such as microalgae, ciliates, amoebae, or cells from tissues of multicellular organisms, since the use of SAGs and RADseq by themselves are not organism-specific and have been used for a variety of species (Lasken and McLean 2014; Andrews et al. 2016; Gawad et al. 2016).

We chose multiple displacement amplification (MDA; Dean et al. 2002; Hosono et al. 2003) for WGA in SAG-RAD because of its high accuracy and extensive genome coverage. MDA generates long DNA products (up to 70 kb) and yields a high quantity of DNA from starting material as small as single-cell genomes. The phi29 polymerase used in the isothermal reaction is characterized by high processivity and high fidelity (error rate of 10^−6^–10^−7^ errors per nucleotide). However, potential coverage bias, including allelic dropout (ADO), and sensitivity to the contamination of nontarget DNA have been reported for MDA (Sabina and Leamon 2015) and had to be assessed to evaluate its use in SAG-RAD.

To investigate which MDA method was most suitable for SAG-RAD, we evaluated three different protocols that employ varied polymerase and priming combinations: 1) Repli-g (Hosono et al. 2003; Qiagen), which is based on phi29 polymerase and random hexamer oligonucleotide primers; 2) WGA-X (Stepanauskas et al. 2017), where the combination of the mutant polymerase EquiPhi29 (Thermo Fisher Scientific) along with random heptamer primers has been found to reduce amplification bias, especially in GC-rich genomes; and 3) TruePrime (Picher et al. 2016; Expedeon), which combines phi29 with a primase instead of random oligonucleotide primers to reduce amplification bias.

Following WGA using the MDA protocol, we prepared single-digest RAD (sdRAD) libraries (Baird et al. 2008). Double-digest RAD of the MDA product has previously been shown to result in adequate libraries but requires relatively high amounts (several nanograms) of starting material (Blair et al. 2015; De Medeiros and Farrell 2018). sdRAD is well suited for organisms with large and complex genomes (Andrews et al. 2016), allows identification and filtering of polymerase chain reaction (PCR) duplicates (Rochette et al. 2019), and has been successfully used in population genetic studies of *G. semen* cultures (Rengefors et al. 2021).

## Results and Discussion

We evaluated the SAG-RAD method using five criteria: genome recovery and coverage (criteria I and II); accuracy of SAG-RAD compared with RADseq from cultured bulk material, that is, not single cells (criteria III and IV); and validation of the method using natural samples (criterion V). Specifically, we assessed: I) the number of loci that were recovered with different WGA protocols and reproducibility for clonal, replicate samples; II) the coverage and uniformity of coverage; III) the concordance with cultured bulk RAD—that is, comparison with bulk RAD samples from cultured material; IV) the recovery of heterozygous loci with SAG-RAD; and V) the ability to resolve the population structure in a set of natural samples and cultured clones.

**Fig. 1.**
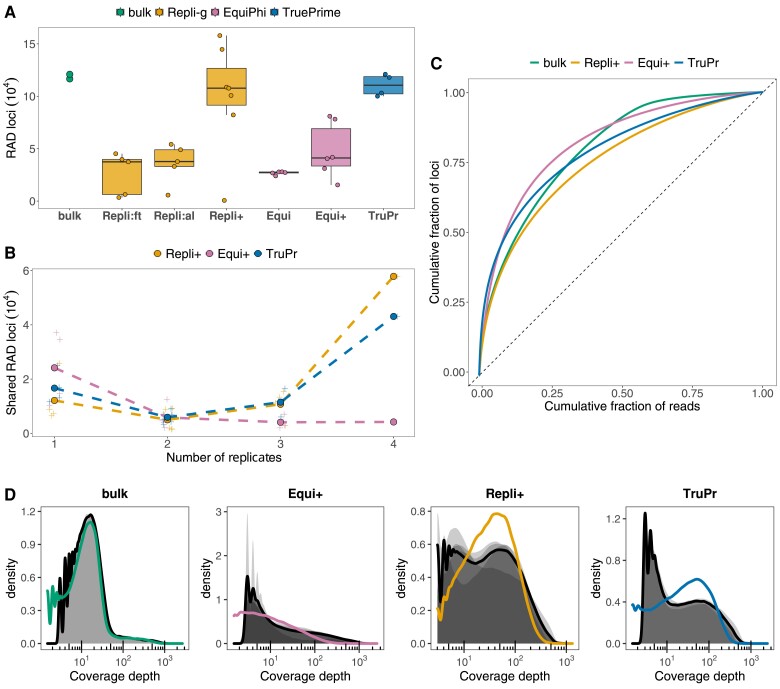
Genome recovery and coverage of SAG-RAD samples compared with bulk RAD samples. (*A*) Number of RAD loci per individual replicate. (*B*) Number of shared loci among replicates. (*C*) Lorenz curves to evaluate coverage uniformity along the genome (cumulative fraction of total reads that cover a given cumulative fraction of loci). (*D*) Density distribution of RAD loci coverage depth—cumulative (black line) and median (colored line).

First, we compared the sequence variants obtained with different MDA protocols to one another and to a bulk RAD sample to assess the extent of genome coverage as the number of recovered loci (criterion I; fig. 1*A*). With Repli-g, we tested the effect of an initial repeated freeze-thaw lysis (Repli:ft) and compared it with alkaline DNA denaturation only, with no preceding lysis step (Repli:al). We further optimized our Repli-g protocol for SAG-RAD by lowering the temperature during alkaline DNA denaturation and by reducing the amplification time (Repli+). Optimization of the Repli-g protocol resulted in a substantial increase in the median number of loci from 37,440 (Repli:ft) and 37,734 (Repli:al) to 107,735 with Repli+, similar to the numbers of both bulk RAD (118,784) and TruePrime (110,514). For EquiPhi29, we compared the effects of long (Equi) and short (Equi+) amplification times. Shorter amplification increased the median number of loci from 27,220 (Equi) to 41,155 (Equi+) with higher variation. Samples with fewer than 10,000 loci were excluded from downstream analyses (see Materials and Methods). In both Repli+ and TruePrime, reproducibility among replicates was high, with the majority of loci shared among two to four replicates (fig. 1*B*), whereas with Equi+, most loci were specific to individual samples. We further analyzed uniformity of coverage (criterion II) using Lorenz curves, plotting the cumulative fraction of total reads that cover a given cumulative fraction of the genome (fig. 1*C*). Here, a perfectly diagonal line corresponds to uniform coverage, whereas deviation from the diagonal is indicative of biased coverage. Repli+ amplifications led to the most uniform coverage. We found the coverage depth distributions of loci (fig. 1*D*) to peak around 10–30× for bulk RAD and around 10–100× for Repli+. Coverage of bulk RAD and Repli+ loci was thus more centered around the optimal coverage depth of >20× (Rochette and Catchen 2017), whereas TruePrime and Equi+ showed a wider distribution that was skewed toward lower coverage depth.

**Fig. 2.**
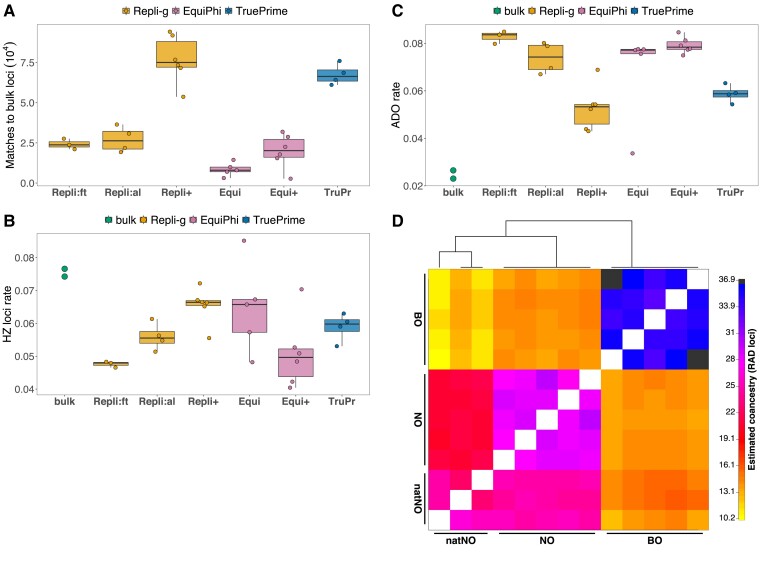
Concordance of SAG-RAD with RAD from bulk samples, recovery of heterozygous loci, and population structure inference from SAG-RAD samples. (*A*) Number of single-cell loci that match loci of bulk RAD samples. (*B*) Proportion of heterozygous (HZ) loci in bulk and SAG samples. (*C*) Rate of loci where ADO is observed in a SAG sample compared with bulk samples. (*D*) Clustered fineRADstrucutre coancestry matrix of cultured isolates from lake Bökesjön, Sweden (BO) and Nobleboro, USA (NO) and natural NO (natNO) isolates (all are SAG-RAD samples).

Assessment of concordance of loci from the different MDA protocols with bulk RAD loci (criteria III and IV) revealed a high overlap with bulk RAD with a median number of 72,097 matching loci in samples amplified with Repli+ and 63,432 matching loci in TruePrime-amplified samples (fig. 2*A*). The number of matches to bulk RAD loci was substantially higher in Repli+ compared with both Repli:al and Repli:ft. The proportion of heterozygous loci (fig. 2*B*) was highest in single-cell samples amplified with Repli+, with a median of 0.067 compared with 0.075 in bulk RAD samples. The proportion of loci that were heterozygous in the catalog of RAD loci created from bulk samples, but homozygous in SAG samples, that is, where one allele was dropped, was lowest in Repli+ samples with a median of 0.053 compared with 0.027 and 0.023 in the two bulk RAD samples (fig. 2*C*). Based on the above results, we concluded that the Repli+ protocol yielded the best results, with the highest number of matched loci compared with the bulk samples while simultaneously showing the highest uniformity of coverage and the lowest frequency of ADO.

Finally, we used the Repli+ protocol to test the ability of the SAG-RAD approach to resolve the population structure in a set of natural samples and cultured clones (criterion V). The analysis of population structure using a clustered coancestry matrix (fig. 2*D*) of cultured isolates from lake Bökesjön (BO), Sweden, and Nobleboro (NO), USA, and uncultured NO cells, revealed higher relatedness of individuals within the three populations than between populations, as expected. The hierarchical structure between the localities was clearly inferred. Three populations were correctly identified, and natural NO cells showed high relatedness with cultured NO samples.

Taken together, we have demonstrated that combining our optimized Repli-g MDA protocol (Repli+) for WGA from single cells with sdRAD enables analysis of population genomics using RADseq from single cells. Using this optimized SAG-RAD approach, we were able to recover a high number of loci across samples, similar to those obtained from RADseq on *G. semen* cultures (Rengefors et al. 2021). Our optimized approach further yielded high uniformity of coverage compared with bulk RAD, as well as a relatively high recovery of heterozygous loci. This was achieved by a lower temperature during DNA denaturation and a shorter amplification time to counter the amplification bias in Repli-g MDA. Although we were able to substantially reduce the loss of heterozygosity using Repli+, ADO rates in SAG-RAD were still higher than in bulk RAD analysis from clonal cultures. This indicates potential for additional optimization to further reduce amplification bias to improve the genotyping accuracy in SAG-RAD. Another potential risk in WGA of single cells is contamination (Woyke et al. 2011), since small amounts of DNA from nontarget species in the amplification reaction can have strong downstream effects. Isolated single cells were washed several times in our study, and we observed only 5–6% contaminant reads in SAG-RAD samples from natural population isolates compared with 8% in bulk extracts from nonaxenic cultures. To ensure recovery of a sufficient number of samples for downstream analyses, more samples than needed should be used in SAG-RAD, to account for potential sample dropout due to a low number of loci being retained in some samples. Our SAG-RAD protocol can be modified for use on other organisms of interest but may require adjustment of conditions for single-cell lysis. For organisms with GC-rich genomes, the use of WGA-X (Stepanauskas et al. 2017) should be considered. SAG-RAD may be further extended to employ other methods for reduced representation sequencing, including different RAD sequencing variants.

In conclusion, the SAG-RAD method enables population genetic and genomic studies on natural populations of microeukaryotes that cannot be cultured, particularly those with large genomes. This novel approach thus allows investigation of genetic diversity, gene flow, adaptation, dispersal, and biogeography in hitherto unexplored species.

## Materials and Methods

### Single-Cell Isolation by Manual Micropipetting

Single cells of *G. semen* were isolated manually under a stereomicroscope (50–80× magnification) using micropipettes custom-made from microcapillaries (minicaps 100 µl; Hirschmann). Isolated cells were washed twice in 2 µl droplets of sterile filtered (0.2 µm) MWC + Se culture medium (Gollnisch et al. 2021) and once in 2 µl droplets of 1× phosphate-buffered saline (PBS) buffer pH 7.4 (Gibco) on sterile 96-well plate lids. Both medium and buffer were decontaminated through UV treatment at 254 nm wavelength using a UV crosslinker (Spectrolinker) before use. Individually washed single cells were transferred into PCR tubes (Sarstedt) in 1 μl PBS, using a new glass capillary for each isolation and each transfer to avoid cross-contamination. All single-cell isolates were frozen immediately and were stored at −80 °C until amplification.

### MDA of SAGs

DNA surface decontaminant (DNA Away) was applied to all surfaces and equipment prior to the preparation of amplification reactions. The preparation of amplification reactions was carried out inside a DNA/RNA UV-cleaner box with a built-in UV recirculator (UVT-B-AR; Biosan), and the entire working area was decontaminated with UV light (wavelength 254 nm) for 45 min before and after each use. All work in the UV-cleaner box was carried out wearing laboratory gloves and Tyvek sleeves (DuPont) to avoid contamination. Different methods for cell lysis and MDA were tested in order to optimize the amplification of single-cell genomes for SAG-RAD.

#### Testing and Optimization of Single-Cell Lysis and MDA

Repli-g (Hosono et al. 2003) amplifications were prepared using the Repli-g Single Cell Kit (Qiagen) following the manufacturer's instructions but using a reduced volume (one-fourth) for each reagent in each step (final reaction volume 12.5 µl). Primers in Repli-g MDA are phosphothioate-modified random hexamers. The temperature for amplification with phi29 polymerase is 30 °C. Inclusion of a lysis step consisting of three freeze-thaw cycles, followed by alkaline DNA denaturation for 10 min at 65 °C, was compared with alkaline DNA denaturation for 10 min at 65 °C without preceding freeze-thaw lysis step. Carrying out alkaline DNA denaturation for 10 min at 20 °C was tested instead of the original 65 °C. A reduced amplification time of 2 h was compared with the original 8 h.

For WGA-X (Stepanauskas et al. 2017) amplifications, EquiPhi29 polymerase (Thermo Fisher Scientific), a thermostable mutant of phi29 polymerase (Povilaitis et al. 2016), was used together with exo-resistant phosphothioate-modified random heptamers (Thermo Fisher Scientific) as primers. The preparation of amplification reactions was modified from the manufacturer's instructions and Stepanauskas et al. (2017) as follows: Alkaline lysis and DNA denaturation were performed by the addition of 0.75 µl lysis buffer consisting of 0.4 M KOH, 10 mM EDTA, and 100 mM DTT to the single-cell isolate in 1 µl PBS. The reaction was incubated at 20 °C for 10 min before adding 0.75 µl stop solution (1 M Tris-HCl, pH 4). DNA was further denatured in a 5 µl reaction containing 0.5 µl reaction buffer (10×), 1 µl exo-resistant random primers (500 µM; Thermo Fisher Scientific), 2.5 µl cell material after lysis, and 1 µl ultra-pure H_2_O. This reaction was incubated at 95 °C for 3 min and then immediately put on ice for 3 min. To prepare the WGA-X amplification reaction, 1.5 µl reaction buffer (10×), 0.2 µl DTT (100 mM), 2 µl dNTP mix (10 mM each), 10.3 µl ultra-pure H_2_O, and 1 µl EquiPhi (10 U/µl) were added to the denatured DNA mix (5 µl). The amplification temperature was 45 °C, and amplification times of 1 and 3 h were compared.

TruePrime (Picher et al. 2016) amplifications were prepared using the TruePrime Single Cell WGA Kit (Expedeon) following the manufacturer's instructions with a reduced volume (½) for each reagent in each step (final reaction volume 12.5 µl). Instead of random oligonucleotide primers, *Tth*PrimPol DNA primase is used in TruePrime to generate primers. The temperature for amplification with phi29 polymerase is 30 °C.

To measure amplification curves during method optimization, SYTO 13 (Invitrogen) nucleic acid stain was added at 0.5 µM final concentration to each amplification reaction by substituting part of the ultra-pure H_2_O (Repli-g and TruePrime: 0.625 µl; WGA-X: 1 µl). Isothermal amplification reactions were carried out on a real-time PCR detection system (CFX96 Touch; Bio-Rad) with a plate read (SYBR channel) every 15 min and a final heat inactivation at 65 °C for 3 min (Repli-g) or 10 min (WGA-X and TruePrime).

The amplification products were purified using a spin column gDNA cleanup kit (Genomic DNA Clean and Concentrator-25; Zymo Research) and eluted in EB buffer (Qiagen). DNA was quantified fluorometrically (Qubit dsDNA BR Assay Kit; Invitrogen) and the size distribution of the amplification products was visualized using agarose gel electrophoresis (1× Tris-acetate-EDTA (TAE) buffer, 1% agarose, 6.5 V/cm, 45 min).

#### Optimized Single-Cell Lysis and MDA Protocol for SAG-RAD

In the optimized Repli-g MDA protocol for SAG-RAD, no freeze-thaw lysis step was employed, and alkaline DNA denaturation was carried out for 10 min at 20 °C. Isothermal amplification reactions were run for 2 h. Late amplifications (higher critical point Cp) were found to recover fewer loci compared with early amplifications (lower critical point Cp) and are thus of minor interest. Extended amplification times could thereby further intensify biased coverage or may result in nonspecific DNA amplification.

### RADseq Library Preparation

The sdRAD library preparation protocol was modified from Amores et al. (2011) and Etter et al. (2011). For each sample, 1 µg of genomic DNA (either SAG or bulk extracted) in EB buffer was digested with 0.5 µl *SbfI*-HF (New England Biolabs) restriction enzyme and 5 µl NEB4 buffer (New England Biolabs) in a 50 µl reaction. The digestion reaction was incubated at 37 °C for 60 min, then inactivated at 80 °C for 20 min and slowly cooled to room temperature for 45 min using a thermocycler (Veriti 96 well; Applied Biosystems).

For P1 sequencing adapter ligation, 1 µl of 10× NEB2 buffer (New England Biolabs), 5 µl ultra-pure water, 3 µl of 100 nM barcoded P1 adapter, 0.6 µl of 100 nM rATP, and 0.5 µl of 200 U/µl T4 DNA ligase (New England Biolabs) were added to each RAD library tube. The ligation reaction was incubated at 20 °C for 60 min, then inactivated at 65 °C for 30 min and slowly cooled to room temperature for 45 min using a thermocycler (Veriti 96 well; Applied Biosystems). The P1 adapters contained unique 7-bp barcodes to allow multiplexing samples in downstream library preparation.

Samples of each method with different P1 adapter barcodes were multiplexed randomly and sheared to a target length of 400 bp using a focused ultrasonicator (M220 with XTU insert; Covaris) following the manufacturer's instructions (130 µl sample volume in microTUBE AFA Fiber Snap-Cap vials). Size selection of the sheared libraries was performed using AMPure XP beads (Beckmann Coulter) on a sample volume of 60 µl. For left-side size selection and to remove P1 adapter dimers, a bead suspension to sample ratio of 0.8 was used to select for DNA fragments longer than 300 bp. Fragments shorter than 600 bp were excluded using a ratio of 0.55 in the right-side size selection step, and the selected fraction was finally eluted from the beads in 22 µl EB buffer.

The Quick Blunting Kit (New England Biolabs) was used for end-repair, and 3′-dA-overhangs were added with Klenow Fragment (3′→5′ exo-; New England Biolabs). Reaction cleanup following each reaction was performed using the MiniElute Reaction Cleanup Kit (Qiagen), with three elution steps instead of one to maximize DNA yield in the eluate. P2 adapters were then ligated to each library. Each P2 adapter ligation reaction was incubated at room temperature for 60 min, followed by reaction cleanup and removal of P2 adapter dimers (left-side size selection with AMPure XP beads as described above for the removal of P1 adapter dimers) and was finally eluted in 45 µl EB buffer.

Concentrations of the resulting library templates were quantified fluorometrically (Qubit dsDNA BR Assay Kit; Invitrogen). Libraries were amplified from 70 ng of template DNA per 100 µl reaction (split into 4 parts during amplification) using Phusion High-Fidelity PCR Master Mix (Thermo Fisher Scientific) and running 18 cycles (30 s 98 °C, 18× [10 s 98 °C, 30 s 65 °C, 30 s 72 °C], 5 min 72 °C, hold 4 °C) on a thermocycler (Veriti 96 well; Applied Biosystems). Following a final reaction cleanup with AMPure XP beads, concentrations of the amplified libraries were quantified fluorometrically (Qubit dsDNA BR Assay Kit; Invitrogen) and sequencing pools were created, combining 40 samples with different P1 adapters in each pool.

### Sequencing

Paired-end sequencing with a read length of 150 bp was performed on an Illumina NovaSeq 6000 equipped with an SP flow cell using v1 sequencing chemistry and 10% PhiX spike-in at the SNP&SEQ Technology Platform of the SciLifeLab facility in Uppsala, Sweden.

### Assembly of RAD Loci

Sequences from all RAD libraries were processed using the software Stacks 2 (Rochette et al. 2019) version 2.53. Stacks process_radtags was used to demultiplex samples based on inline barcodes (allowing for one mismatched base in the barcode) and to filter reads. Reads that contained adapter sequences (allowing for two mismatched bases), reads with an uncalled base, reads with low-quality scores, and reads with no intact *Sbf*I restriction enzyme cut site were discarded. All samples were then filtered to exclude potential human or bacterial contaminant reads using the taxonomic sequence classifier Kraken 2 (Wood et al. 2019) version 2.0.8-beta.

**Fig. 3.**
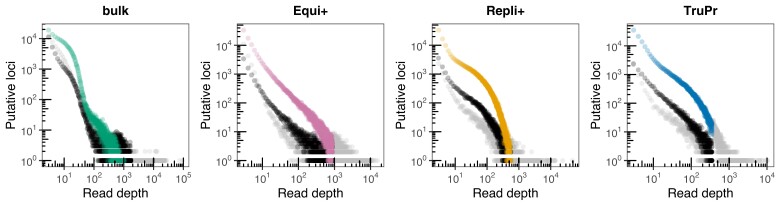
Read depth of putative loci in SAG-RAD samples compared with bulk RAD samples. Number of putative loci (in colors), confounded loci (consisting of too many putative variants; in gray) and putative loci with extreme coverage (>mean + 3 × standard deviation; in black) of different read depths.

Sample loci were built and analyzed de novo using the Stacks programs ustacks to build putative loci (stacks), cstacks to create a catalog of loci comprising individuals from all lakes, and sstacks to match sample loci against the catalog. Parameters in ustacks were set to a minimum stack depth of 3 (parameter m) and a distance allowed between stacks of 2 (parameter M) to maximize the number of utilized reads and polymorphic SNPs while maintaining a mean coverage of at least 20×. Sites with extreme coverage (>mean + 3 × standard deviation) and sites with more than three stacks (confounded loci) were filtered out (fig. 3). In general, both are likely from repetitive genomic regions. In SAG-RAD, they can be a result of biased amplifications. The catalog was created allowing for two mismatches between sample loci (parameter n). All samples were then matched back against the catalog using sstacks. Paired-end reads were then incorporated to assemble contigs using Stacks tsv2bam and Stacks gstacks, and PCR duplicates were filtered based on insert length.

### Data Processing and Analysis of RAD Loci

Data processing and analysis were conducted using Python 3 and R (R Core Team 2020) version 3.6.3 with the additional packages tidyverse 1.2.1, UpSetR 1.4.0, Rmisc 1.5, ggpubr 0.2.1, Cairo 1.5–12.2, and scales 1.1.1. The number of RAD loci per sample was determined as the total number of distinct loci identified in Stacks ustacks. Heterozygous loci were defined as loci with more than one allele. Samples with fewer than 10,000 RAD loci were excluded from downstream analysis, keeping 5/7 Repli+ samples, 4/5 Repli:al samples, 4/6 Repli:ft samples, and 4/4 TruPr samples. To identify congruence of SAG-RAD loci and bulk RAD loci, SAG-RAD samples were matched against the catalog of bulk RAD samples using Stacks sstacks. The rate of ADO was determined as the number of homozygous loci that matched diploid catalog loci (loci with two alleles in the bulk RAD catalog) relative to the total number of matched loci. A clustered coancestry matrix of cultured isolates from lake Bökesjön (BO) and Nobleboro (NO), and natural NO isolates was created in fineRADstructure (Malinsky et al. 2018), using loci that are present in all individuals of all three populations.

### Cell Culture of Monoclonal *G. semen* Strains

Cultures of monoclonal *G. semen* strains were established from samples collected at lake Bökesjön (Sweden; 55.576 N, 13.437 E) in 2010 (strain BO-182) and at Damariscotta Lake near Nobleboro (ME, USA; 44.104 N, 69.474 W) in 2015 (strain NO-018). Single cells of *G. semen* were isolated manually under a stereomicroscope (50–80× magnification) using micropipettes custom-made from microcapillaries (minicaps 100 µl; Hirschmann). The isolates were transferred into a 1:1 mixture of 0.2 µm sterile filtered water from the respective lake and MWC + Se medium to initiate the monoclonal cultures, which were later grown in a pure MWC + Se medium. Culturing success rates (percentage of survival in culture) were 49% (21–75% during April–October 2009; 49% on July 16, when strain BO-182 was isolated) for isolates from lake Bökesjön (Lebret et al. 2012) and 21% for isolates from Damariscotta Lake. The culture medium (MWC + Se) consisted of a modified WC medium (Guillard and Lorenzen 1972) complemented with 1.196 µg/l Na_2_SeO_3_.5H_2_O and contained TES instead of TRIS buffer at a final concentration of 115 mg/l. The isolated strains were kept in a climate chamber at a temperature of 20 °C and 40 μmol photons/m^2^s light intensity on a 12:12 h light:dark cycle.

### DNA Extraction for Bulk RAD From *G. semen* Cultures

When sufficient biomass had been grown (∼150,000 cells), cultures of *G. semen* strains BO-182 and NO-018 were harvested by centrifugation, the supernatant was removed, and pellets were stored at −80 °C. DNA extractions were made by CTAB extraction according to Lebret et al. (2012), resuspending the extracted DNA in EB buffer (10 mM Tris-Cl, pH 8.5) instead of TE buffer in the final step of the protocol. RADseq libraries were prepared from the extracted DNA of bulk culture samples as described above.

### Natural Population Sample Collection

Another sample of a *G. semen* population was collected from Damariscotta Lake near Nobleboro (ME, USA) in 2018 for downstream SAG-RAD. The sample was collected using a plankton net (mesh size 20 µm) and filtered through a 150-µm mesh to exclude larger grazers. Cells of *G. semen* were enriched in a droplet of 0.2 µm sterile filtered lake water by manual micropipetting and were isolated from the enriched sample as described above.

## Data Availability

Sequence data are available through the NCBI SRA database (BioProject ID PRJNA891215): https://www.ncbi.nlm.nih.gov/bioproject/PRJNA891215.
